# Cardioprotective efficacy depends critically on pharmacological dose, duration of ischaemia, health status of animals and choice of anaesthetic regimen: a case study with folic acid

**DOI:** 10.1186/s12967-014-0325-8

**Published:** 2014-11-29

**Authors:** Coert J Zuurbier, Andre Heinen, Anneke Koeman, Roy Stuifbergen, Theodorus BM Hakvoort, Nina C Weber, Markus W Hollmann

**Affiliations:** Laboratory of Experimental Anaesthesiology, Department of Anaesthesiology, Academic Medical Centre, Amsterdam, The Netherlands; Institute of Cardiovascular Physiology, Heinrich-Heine University, Düsseldorf, Germany; Tytgat Institute for Liver and Intestinal Research, Academic Medical Centre, Amsterdam, The Netherlands; Department of Anaesthesiology, Academic Medical Centre, University of Amsterdam, Meibergdreef 9, Amsterdam, 1105 AZ The Netherlands

**Keywords:** Ischaemia/reperfusion, Translational science, eNOS, Infarct, Cardioprotection, Aged, Diabetic, Anaesthesia, Propofol, Pentobarbital

## Abstract

**Background:**

Acute, high-dose folic acid (FA) administration has recently been shown to possess unprecedented effective cardioprotection against ischaemia/reperfusion (I/R) injury. Here we explore the translation potential of FA as treatment modality for cardiac I/R.

**Methods:**

Dependency of FA protection on dose, ischaemia duration, and eNOS was examined in an isolated mouse heart I/R model, whereas dependency on animal health status and anaesthesia was examined in an *in vivo* rat model of regional cardiac I/R.

**Results:**

50 μM FA provided maximal reduction (by 95%) of I/R-induced cell death following 25 min ischaemia in isolated wild-type hearts, with protection associated with increased coupled eNOS protein. No protection was observed with 35 min I or in eNOS^−/−^ hearts. Acute intravenous administration of FA during a 25 min ischaemic period reduced infarct size by 45% in *in vivo* pentobarbital-anaesthetised young, healthy rats. FA did not reduce infarct size in aged or pre-diabetic rats, although it did preserve hemodynamics in the pre-diabetic rats. Finally, using a clinically-relevant anaesthetic regimen of fentanyl-propofol anaesthesia, FA treatment was ineffective in young, aged and pre-diabetic animals.

**Conclusions:**

The protective potential of an initially promising cardioprotective treatment of high dose FA against cardiac I/R infarction, is critically dependent on experimental conditions with relevance to the clinical condition. Our data indicates the necessity of expanded pre-clinical testing of cardioprotective interventions before embarking on clinical testing, in order to prevent too many “lost-in-translation” drugs and unnecessary clinical studies.

## Background

The past two decades have witnessed the unveiling of many extra- and intracellular pathways and signaling molecules which modulate ischaemia/reperfusion (I/R) injury in a laboratory setting. This intensified search for cardioprotective signals was largely instigated by the discovery of the ischaemic preconditioning phenomenon [1], which demonstrated the presence of endogenous cardioprotective pathways with the potential for pharmacological intervention. Current thinking has been that the next step which needs to be taken is the application of the impressive laboratory knowledge to the clinical arena. However, this step has come with great disappointments. Many laboratory derived cardioprotective interventions have ultimately failed clinically [2,3]. These failures urge the research community to critically examine the possible reasons underlying these failures. Similar disappointing results for translational clinical science are encountered in different disease models [4,5]. A main reason for this poor translation is that there is a lack of incentive for the researcher to report affirmative or negative data, because especially so-called high-impact journals prefer positive studies. This prerequisite of positive and novel results creates a research environment wherein the pre-clinical researcher fine-tunes the research model such that it provides the largest mandible potential for positive results. In the area of cardiac I/R this usually translates into the use of ischaemic periods between 15–30 min, healthy and young animals, pentobarbital anaesthesia and an optimal dosage/timing/administration of the investigated cardioprotective intervention. However, the clinical condition obviously deviates from these preclinical optimal conditions. It is therefore likely that the poor translation of laboratory research into the clinical arena is, at least partly, caused by the neglect of the unglamorous, but necessary, work of full characterisation of the animal model towards the clinical condition, which neglect is driven by the current positive-based research environment [4,5].

In the present work we therefore specifically examine consequences of clinical deviations (ischaemic duration, anaesthetic regimen, health status of the animals) from these optimal laboratory conditions using a novel, highly promising, cardioprotective strategy of high-dose folic acid (FA) treatment against acute cardiac I/R injury [6]. High-dose folic acid was demonstrated to have a very strong protective effect against I/R cardiac damage in the laboratory setting, suggesting an almost curing of heart attacks [7]. These clinical deviations were previously shown to impact other cardioprotective interventions such as various “conditioning (remote, pre- and post-)” and helium interventions [8-11]. In addition, we examined the dose dependency of FA on cardioprotection, and studied to what extent eNOS and its dimeric state are mandatory for FA cardioprotection, as was suggested by [6]. In the present study we hypothesize that FA cardioprotective effects against cardiac I/R injury are critically dependent on: 1) FA dose, 2) ischaemic duration, 3) eNOS, 4) anaesthetic regimens, and 5) health status of the animal.

## Methods

### Animals

C57BL6 wild-type (WT) mice were obtained from Charles River and the eNOS (NOS3)^−/−^ mice [12] were obtained from an in-house bred population. Experiments were performed with male mice at 10–16 weeks of age. Young (2–4 months) and old (>20 months) RccHan:WIST rats, and the Zucker Obese rats (HsdOla: Zucker-Lepr) (3–4 months) were obtained from Harlan. Mice and rats were fed a standard chow (CRM (E) diet, SDS, Witham, England) *ad libitum.* Animals were treated according to the guidelines of the Declaration of Helsinki and all procedures were in accordance with the requirements of the Animal Welfare and Use Committee of the Academic Medical Center of Amsterdam.

### Isolated mouse heart perfusion

In vitro I/R protocol was performed in isolated mouse hearts as previously described with slight modifications [13,14]. Mice were heparinized (15 IU) and anaesthetised with Nembutal (80 mg kg^−1^). After a tracheotomy, the mice were mechanically ventilated and a thoracotomy was performed via midline incision. The aorta was cannulated in situ and myocardial perfusion started before excision of the heart. Hearts were Langendorff-perfused at a constant flow (initial perfusion pressure 80 mm Hg) at 37°C with Krebs-Henseleit solution containing (mmol l^−1^) NaCl 118, KCl 4.7, CaCl_2_ 2.25, MgSO_4_ 1.2, NaHCO_3_ 25, KH_2_PO_4_ 1.2, EDTA 0.5 and glucose 11, gassed with 95% O_2_/5% CO_2_. The perfusate was in-line filtered by a 0.45-μm filter. End-diastolic pressure (EDP) was set at ~4-8 mmHg using a water-filled polyethylene balloon inserted into the left ventricular (LV) cavity via the mitral valve. The hearts were continuously submerged in 37°C perfusate. LV developed pressure was calculated as the systolic pressure (Psys) minus the end-diastolic pressure (EDP). The rate-pressure product (RPP) was the product of the developed LV pressure and the heart rate.

### Experimental protocol isolated hearts

Following a stabilisation period of 20 min, the experimental protocol was started with 35 min normoxic perfusion, followed by 25 min or 35 min ischaemia, and 45 min reperfusion. Hearts were treated with FA by switching to a FA-containing perfusate at t = 5 min of normoxic perfusion for the remaining of the protocol. We performed two different FA cardioprotective dose-finding series with 4 groups each, one dose-finding series for 25 min I (n = 3 per group) and one dose-finding series for 35 min I (n = 3-6 per group). The relatively short period of ischaemia (25 min) is commonly used in laboratory research directed at examining cardioprotective interventions (e.g. [15-17]). Following the establishment of the FA dose–response curve for this short period of ischaemia, a second series of 4 groups was performed to examine this FA dose–response for a more prolonged period of ischaemia (35 min). Finally, in a third series of experiments we examined whether eNOS is mandatory for FA cardioprotective effects. To this end we compared wild-type heart with eNOS^−/−^ hearts (n = 7 per group), using 25 min I and the optimal dose of FA derived from the first series of experiments. The venous effluent was collected at fixed times throughout reperfusion (at 5, 10, 15, 30 and 45 min reperfusion) for determination of lactate dehydrogenase (LDH) leakage as index of necrosis. At the end of the experiments of series 1 and 2, the heart was weighted, submerged in 1 ml homogenisation buffer medium (in mM: 250 sucrose, 20 Hepes (pH 7.4), 10 KCl, 1.5 MgCl2, 1 EDTA, 0.1 PMSF, 5 μg/ml leupeptin, 5 μg/ml aprotinin and 1 μg/ml pepstatin) and homogenized. The homogenate was stored at −80°C until analysis for eNOS dimers as index of eNOS uncoupling.

### Biochemical determinations

LDH activities were determined according to standard spectrophotometric techniques [18]. Dimerization of eNOS was determined by low-temperature electrophoresis in heart homogenates. In short, the homogenate was treated with 0.5% triton and centrifuged at 10.000 g to pellet non-dissolved fragments. Protein concentration was determined in the supernatant by the Bradford method, and the supernatant samples were mixed in a 1:1 dilution with loading buffer, without the addition of mercaptoethanol and without boiling of samples. Samples were loaded on a 6% SDS-PAGE, electrophoresis performed at 4°C and transferred to Immobilon-P Transfer Membranes (Millipore corp). Membranes were incubated overnight with eNOS antibody (1:1000; Cell Signalling). Proteins were detected using chemiluminescence, bands visualized by exposure to photographic film, and densitometry performed. Densities of the dimer eNOS (280 kDa) in the FA-treated groups are expressed relative to the density of eNOS dimer of the control, no-FA treated, group on the same blots (n = 3 hearts per experimental group; each heart measured in triplo).

### *In vivo* cardiac I/R rats

Surgical preparation was performed as reported previously [11]. In short, following pentobarbital anaesthesia (70 mg/kg, i.p.), rats were intubated and mechanical ventilated, and respiratory rate was adjusted to maintain partial pressure of carbon dioxide using in-line capnography within physiological limits. Body temperature was monitored by a rectal probe and maintained at 37°C by the use of a heating pad. The right jugular vein was cannulated for fluid administration (saline plus 20 mM bicarbonate at a rate of 10 ml/kg/h), and the left carotid artery was cannulated for measurement of aortic pressure. The tail vein was cannulated for administration of anaesthesia and FA solution. A lateral left-sided thoracotomy was performed, and a ligature (5–0 Prolene) was passed below a major branch of the left coronary artery. Aortic pressure was digitized using an analog-to-digital converter (PowerLab/8SP; ADInstruments Pty Ltd, Castle Hill, Australia) at a sampling rate of 500 Hz and was continuously recorded on a personal computer using Chart for Windows version 5.0 (ADInstruments).

### Experimental protocol *in vivo* I/R

Following instrumentation, an arterial blood sample was obtained for blood gas and glucose analysis (Cobas b123 POC system, Roche diagnostics) and insulin determination (Mercodia Ultrasensitive Rat Insulin ELISA), following which the animals were left untreated for 20 min before the start of the respective experimental protocol. FA treatment effects were examined in two different series of experiments: one using pentobarbital anaesthesia and the other using fentanyl/propofol anaesthesia. FA effects were examined in young animals, in old animals, and in the pre-diabetic, Zucker obese animals (n = 6-9 animals per group) within each series of experiments. To this end, FA was administered by acute intravenous delivery starting 10 min after the onset of ischaemia. FA (Sigma F8798) was dissolved in bicarbonate-buffered saline solution (5 mg FA/ml saline) and infused during ischaemia (from 10 min I to 20 min I) at a dose of 3.3 mg/100 g BW. Control animals were perfused with an equal volume of saline. Therefore, a total of 6 experimental groups were examined within each anaesthetic regimen. Induction anaesthesia in all rats was achieved with 70 mg/kg pentobarbital. Subsequently, maintenance anaesthesia consisted of pentobarbital (30 mg/kg/h) for the pentobarbital groups, and of fentanyl (1 mg/kg/h) and propofol (8–12 mg/kg/h) for the fentanyl/propofol groups, respectively. Fentanyl-propofol anaesthesia is examined because this regimen is one of the most frequently used in these perioperative, surgical, conditions for which cardioprotective strategies are being developed (e.g. CABG procedures).

### Infarct size measurement

After 120 min of reperfusion, the heart was excised, with the occluding suture left in place, and then mounted on a modified Langendorff apparatus for perfusion with ice-cold normal saline via the aortic root to wash out intravascular blood. After 5 min of perfusion, the coronary artery was reoccluded, and the remainder of the myocardium was perfused through the aortic root with 0.2% Evans blue in normal saline for 10 min. Intravascular Evans blue was then washed out by perfusion with normal saline for 10 min. This treatment identified the area at risk as unstained. The heart was then cut into 2-mm-thick transverse slices. The slices were stained with 0.75% triphenyltetrazolium chloride solution for 10 min at 37°C and fixed in 4% formalin solution for 24 h at room temperature. The area of risk and the infarcted area were determined by planimetry using Image J software by one person that was blinded to the specific treatments of each heart.

### Statistics

All data are presented as means ± SEM. Baseline cardiac parameters of isolated hearts for all groups were compared by one-way ANOVA. One-way ANOVA with Dunnett’s post hoc test was used to compare several FA doses vs no FA in isolated mouse heart experiments, and to compare plasma metabolic parameters of old and Zucker obese animals vs young animals. Student’s t-test were used to compare within group FA treatment effects on infarction. Differences in hemodynamics (MAP, HR) between non-FA and FA-treated groups were analysed by a two-way ANOVA (main effects: FA treatment and experimental time (baseline vs. reperfusion; interaction effects: FA treatment × experimental time) for repeated neasurements followed by contrast comparison. P values <0.05 were considered statistically significant.

## Results

### FA treatment effects in isolated mouse hearts

**Figure 1:**
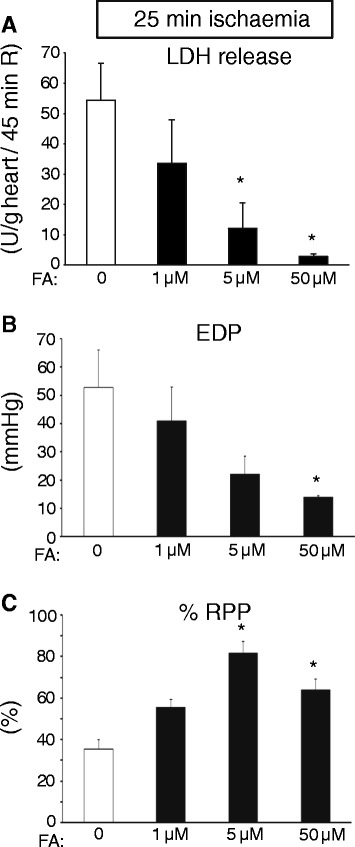
**Increased protection against 25 min I and 45 min R with increasing doses of FA in isolated perfused mouse hearts. (A)** Cumulative lactate dehydrogenase (LDH) release during 45 min R (index of cell death) in groups treated with different FA doses; **(B)** End-diastolic pressure (EDP) at end R for the different groups of FA treatment; **(C)** % Rate-pressure product (RPP) determined at end R and normalized to baseline, pre-ischaemic, values for the different groups. (n = 3 hearts for all groups). Mean ± SEM, * P < 0.05 vs. FA = 0 group.

**Figure 2:**
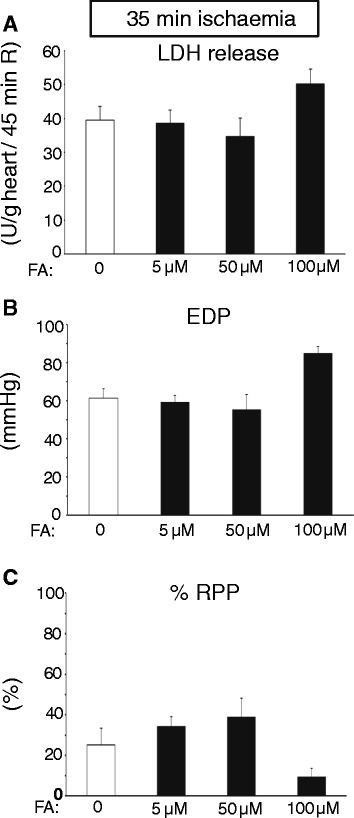
**No protection against 35 min I and 45 min R with increasing doses of FA in isolated perfused mouse hearts. (A)** Cumulative lactate dehydrogenase (LDH) release during 45 min R (index of cell death) in groups treated with different FA doses; **(B)** End-diastolic pressure (EDP) at end R for the different groups of FA treatment; **(C)** % Rate-pressure product (RPP) determined at end R and normalised to baseline, pre-ischaemic, values for the different groups. (n = 3-6 hearts per group). Mean ± SEM, * P < 0.05 vs. FA = 0 group.

Baseline cardiac physiological parameters of the isolated WT hearts (Flow = 11.3 ± 0.4 ml/min/g; EDP = 6.1 ± 0.4 mmHg, Psys = 109 ± 3 mmHg, HR = 367 ± 8 beats/min, and RPP = 37.5 ± 1.3 10^3^ mmHg/min) were similar between groups (Table 1). We first set out to examine to what extent various concentrations of FA were able to protect against a relatively short period of total, global ischaemia (25 min) in the isolated mouse heart (Figure 1). Increasing the FA concentration in the perfusate was associated with a continuous decreases in LDH release (Figure 1A). At the highest FA dose of 50 μM, LDH release was decreased by almost 95%, indicating very effective cardioprotection by FA in this specific model. These FA-induced reductions in cell death were mimicked by improvements of cardiac function: I/R-induced elevation of EDP was reduced by 73%, whereas %RPP recovery increased by 81% with 50 μM FA (Figure 1B and C). Subsequently, FA protective effects were studied employing an extended period of ischaemia (35 min). No protection against I/R-induced LDH release was observed with the various doses of FA (Figure 2A). Increasing FA concentration from 50 μM to 100 μM was even associated with a non-significant increase in LDH release. FA did not improve cardiac function after 35 min I, with a trend towards poorer recovery of the mechanical parameters when FA was increased from 50 μM to 100 μM (Figure 2B and C). Thus, the effect of FA on cardiac I/R injury is critically dependent on FA concentration and duration of the ischaemic insult.

**Table 1:** **Baseline parameters of isolated heart experiments**

**Group**	**Flow (ml/min/g)**	**EDP (mmHg)**	**Psys (mmHg)**	**Heart rate (beats/min)**	**RPP** **(10** ^**3**^ **mmHg/min)**
WT, 25 min I, 0 FA	12.7 ± 0.3	8.0 ± 0.7	99 ± 9	366 ± 3	33.4 ± 3.3
WT, 25 min I, 1 FA	10.9 ± 0.2	8.0 ± 0.8	91 ± 2	415 ± 27	34.3 ± 1.6
WT, 25 min I, 5 FA	9.2 ± 0.3	7.0 ± 0.4	95 ± 6	364 ± 14	32.1 ± 3.2
WT, 25 min I, 50 FA	10.2 ± 0.7	7.3 ± 0.2	102 ± 4	366 ± 24	35.1 ± 3.5
WT, 35 min I, 0 FA	12.0 ± 0.5	4.4 ± 0.7	119 ± 4	356 ± 12	40.6 ± 1.9
WT, 35 min I, 5 FA	12.4 ± 1.0	6.0 ± 0.3	114 ± 4	346 ± 26	36.8 ± 1.7
WT, 35 min I, 50 FA	11.7 ± 1.4	5.0 ± 1.1	121 ± 7	382 ± 9	44.2 ± 2.9
WT, 35 min I, 100 FA	10.1 ± 0.7	5.7 ± 1.2	107 ± 3	347 ± 20	35.6 ± 3.2
eNOS^−/−^, 25 min I, 0 FA	12.7 ± 1.4	5.1 ± 1.9	113 ± 6	383 ± 24	41.8 ± 4.8
eNOS^−/−,^ 25 min I, 50 FA	11.0 ± 1.4	5.2 ± 0.6	107 ± 6	356 ± 29	36.7 ± 3.9

WT, wild-type; 0-1- 5-50-100 FA, 0-1-5-50-100 μM Folic Acid; ml/min/g, ml perfusate per min per g heart wet weight; EDP, end-diastolic pressure; Psys, peak systolic pressure; RPP, rate pressure product. No differences among groups were detected. Mean ± SEM.

### FA protective effects are mediated through eNOS

**Figure 3:**
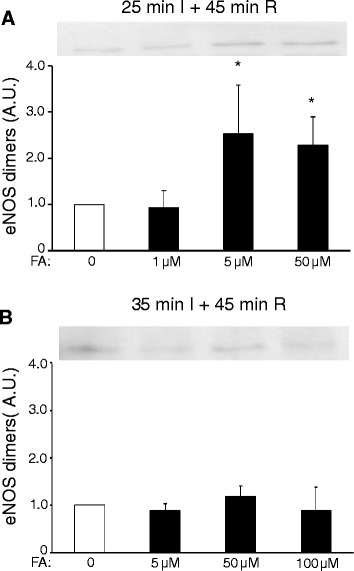
**High-dose FA treatment increases cardiac dimerization of eNOS as compared to no FA treatment following 25 min I and R, but not following 35 min I and R in isolated perfused mouse hearts. (A)** 25 min I: representative Western blot and summary of densitometry analysis, data normalized to FA = 0 group; **(B)** 35 min I: representative Western blot and summary of densitometry analysis, data normalized to FA = 0 group; (n = 3 hearts per group). Mean ± SEM, *P < 0.05 vs. FA = 0 group.

**Figure 4:**
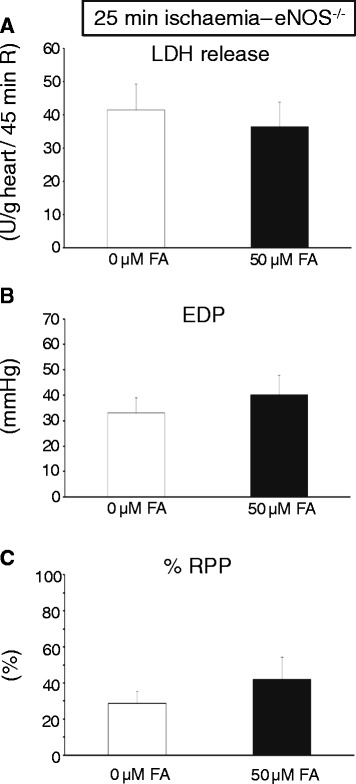
**No protection against 25 min I and 45 min R with FA in eNOS** ^**−/−**^ **-isolated perfused mouse hearts. (A)** Cumulative lactate dehydrogenase (LDH) release during 45 min R (index of cell death) in groups treated with different FA doses; **(B)** End-diastolic pressure (EDP) at end R for the different groups of FA treatment; **(C)** % Rate-pressure product (RPP) determined at end R and normalised to baseline, pre-ischaemic, values for the different groups. (n = 7 hearts per group). Mean ± SEM.

It has been suggested that FA protective effects are mediated through increased coupling of eNOS [6,19]. We therefore examined eNOS dimers at the end of reperfusion in the two series of isolated hearts with different duration of ischaemia (Figure 3). For the 25 min I groups, high dose FA was indeed associated with elevated eNOS dimers as compared to the I/R hearts that received no FA (Figure 3A), whereas no such effect was observed for the 35 min I hearts (Figure 3B). Subsequently, we examined whether the most effective FA dose (50 μM) for cardioprotection against 25 min I in wild-type hearts was effective in eNOS^−/−^ hearts (Figure 4). Baseline cardiac physiological parameters of the isolated eNOS hearts (Flow = 11.7 ± 0.9 ml/min/g; EDP = 5.1 ± 0.9 mmHg, Psys = 108 ± 4 mmHg, HR = 368 ± 16 beats/min, and RPP = 38.5 ± 2.9 10^3^ mmHg/min) were similar between the two groups (Table 1). FA was ineffective against I/R injury in eNOS ^−/−^ hearts, both for LDH release (Figure 4A), EDP (Figure 4B) and %RPP (Figure 4C). These data suggest that FA protective effects against cardiac I/R injury are mediated through the eNOS enzyme, presumably by keeping this enzyme in a coupled (= dimer) state.

### FA treatment effects against *in vivo* cardiac I/R in pentobarbital-anaesthetised rats

**Figure 5:**
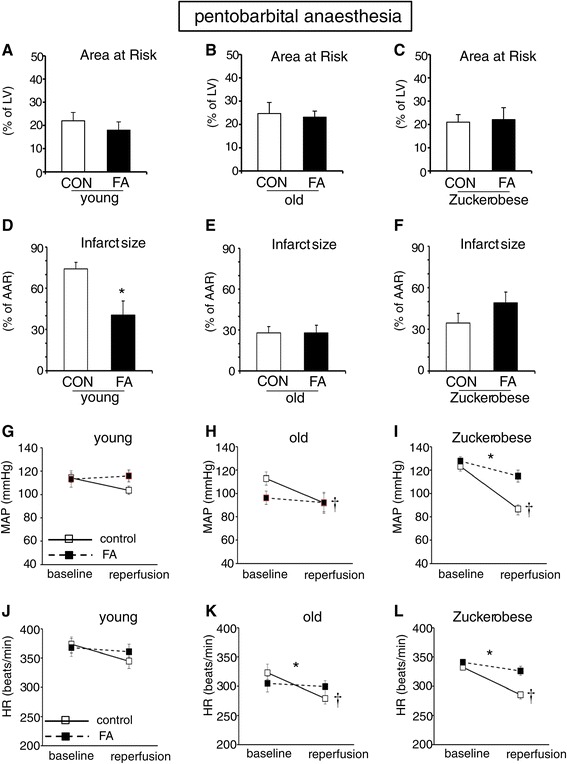
**FA effects against** ***in vivo*** **cardiac LAD ischaemia-reperfusion in pentobarbital-anaesthetised rats.** FA treatment during ischaemia reduced infarction in young, but not in old or Zucker obese animals. **(A-F)**: Area at risk as percentage of left ventricle and Infarct size as percentage of area at risk (AAR) for young **(A, D)**, old **(B, E)** and Zucker obese **(C, F)** rats; **(G-I)**: Mean arterial pressure (MAP) at baseline and end rperfusion for young **(G)**, old **(H)** and Zucker obese **(I)** rats; **(J-L)**: Heart rate (HR) at baseline and end reperfusion for young **(J)**, old **(K)** and Zucker obese **(L)** rats. (n = 6-10 animals per group). Mean ± SEM, *P < 0.05 vs. FA = 0 group for infarct size; † P < 0.05 baseline value vs reperfusion value for control group; *P < 0.05 for interaction effect (experimental time effect (baseline minus reperfusion) for control vs. that for FA treated group. Mean ± SEM.

To determine whether FA treatment may also be beneficial in several *in vivo* conditions of cardiac I/R, acute FA administration was studied in an *in vivo* cardiac regional I/R model. We first examined FA protection in healthy and diseased animals anaesthetised with pentobarbital, an anaesthetic frequently used in animal research (e.g. [6]). The Zucker obese animals were borderline hyperglycemic with significantly elevated insulin levels, indicating the pre-diabetic condition of these animals in the present study (Table 2). The area at risk for the various groups was 22.0 ± 3.6%, 24.7 ± 2.7%, and 21.0 ± 3.2% for young, old and Zucker obese animals, respectively, and was similar for the non-FA and FA treatment groups (Figure 5A-C). Acute intravenous FA administration during the 25 min ischaemic period reduced infarct size in pentobarbital-anaesthetised young animals from 74.2 ± 4.8% in controls to 40.6 ± 10.4% (Figure 5D). In contrast to young animals, acute administration of FA did not reduce infarct size in pentobarbital-anaesthetised aged rats (Figure 5E) or pre-diabetic, Zucker obese rats (Figure 5F). FA treatment effects on changes in haemodynamic data, going from baseline to end reperfusion, are displayed in Figure 5G-I for MAP, and in Figure 5J-L for HR. In general, non-FA treated hearts for the aged and Zucker obese all displayed a decrease in these haemodynamic parameters following the cardiac I/R insult. FA treatment seems to attenuate these decreases in haemodynamic parameters, with significant FA effects for MAP in Zucker obese animals, and for HR in aged and Zucker obese animals. In summary, in pentobarbital-anaesthetised animals, acute FA administration during ischaemia reduced infarction in young, healthy animals, but not in aged or pre-diabetic animals. There is a non-significant trend in improved haemodynamics with FA treatment in pentobarbital-anaesthetised animals, which becomes significant in aged and pre-diabetic animals.

**Table 2:** **Baseline blood glucose and insulin values of the different experimental groups**

	**Pentobarbital**		**Propofol/Fentanyl**	
**Animals**	**Glucose (mM)**	**Insulin (ng/ml)**	**Glucose (mM)**	**Insulin (ng/ml)**
Young (2–4 mo)	6.7 (0.1)	2.6 (0.2)	6.3 (0.2)	2.6 (0.4)
Old (>20 mo)	5.9 (0.3)	5.0 (0.9)	6.0 (0.3)	5.4 (0.9)
Zucker (3–4 mo)	7.8* (0.4)	22.9* (1.1)	7.1 (0.5)	19.8* (1.5)

*< p <0.05 vs Young.

### FA treatment effects against *in vivo* cardiac I/R in fentanyl/propofol-anaesthetised rats

**Figure 6:**
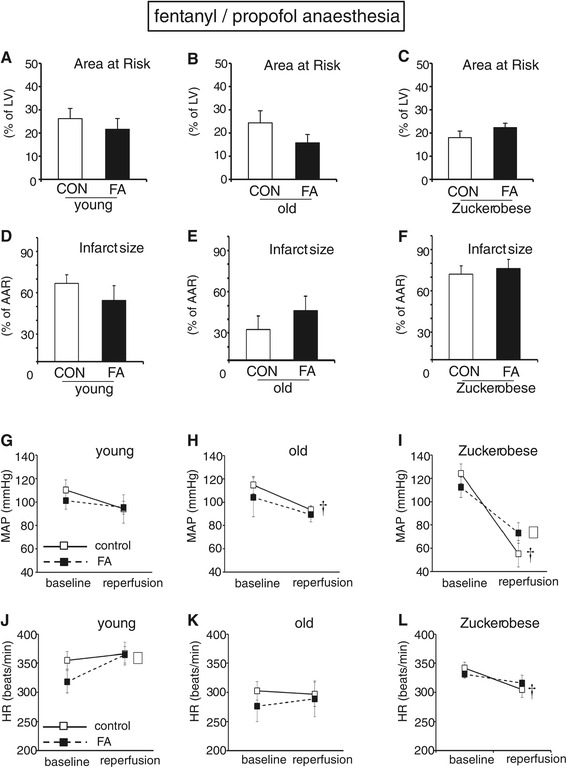
**FA treatment was without effect against** ***in vivo*** **cardiac LAD ischaemia-reperfusion in fentanyl/propofol-anaesthetised rats.** FA treatment during ischaemia did not affect infarction in young, old or Zucker obese animals. **(A-F)**: Area at risk of percentage of left ventricle and infarct size as percentage of area at risk (AAR) for young **(A, D)**, old **(B, E)** and Zucker obese **(C, F)** rats; **(G-I)**: Mean arterial pressure (MAP) at baseline and end reperfusion for young **(G)**, old **(H)** and Zucker obese **(I)** rats; **(J-L)**: Heart rate (HR) at baseline and end reperfusion for young **(J)**, old **(K)** and Zucker obese **(L)** rats. (n = 6-8 animals per group). † P < 0.05 baseline value vs reperfusion value for control group; # P < 0.05 baseline value vs reperfusion value for FA group. Mean ± SEM.

In order to facilitate the translational aspect of FA treatment, we also examined the effectiveness of FA using a clinically-relevant anaesthetic regimen of fentanyl/propofol in healthy and diseased animals (Figure 6). The area at risk for the different fentanyl/propofol-anaesthetised groups was 26.2 ± 4.4%, 24.4 ± 5.2%, and 18.2 ± 2.8% for young, old and Zucker obese animals, respectively, and was similar for the non-FA and FA treatment groups (Figure 6A). Acute FA administration during the ischaemic period was unable to reduce infarct size in fentanyl/propofol-anaesthetised young, healthy animals (Figure 6D). Moreover, FA treatment also did not reduce infarction in the aged (Figure 6E) and pre-diabetic animals (Figure 6F). The I/R-induced changes in haemodynamic parameters, going from baseline to end reperfusion, for MAP (Figure 6G-I) and HR (Figure 6J-L) with fentanyl/propofol anaesthesia also demonstrated a decrease during the experiment, as was observed for the pentobarbital-anaesthetised animals. However, in contrast to the pentobarbital-anaesthetised groups, FA treatment was now unable to significantly attenuate these haemodynamic decreases in any of the fentanyl/propofol-anaesthetised groups.

## Discussion

The major findings of this study are the following: 1) the cardioprotective potential of a high-dose folic acid against cardiac I/R injury is critically dependent on the FA dose and the duration of ischaemia, 2) FA protection is mediated through eNOS, probably through maintenance of the enzyme dimeric configuration, 3) FA offers no protection against cardiac infarction in aged or pre-diabetic animals in an *in vivo* model of regional cardiac I/R, 4) FA cardioprotective potential is lost with a fentanyl/propofol anaesthetic regimen, and 5) FA attenuates I/R-induced decrease in hemodynamics with pentobarbital anaesthesia, which FA effect on hemodynamics is lost with fentanyl/propofol anaesthesia.

### Folic acid protection and FA concentration

The present study showed that cardioprotection against I/R injury induced by 25 min of ischaemia in the isolated mouse heart began at a concentration of 5 μM FA and was further increased with 50 μM, whereas 1 μM was not associated with significant cardioprotection. The protective concentrations were associated with increased eNOS coupling. Moens *et al.* [6] also demonstrated cardioprotection with 4.5 μM FA in unloaded, isolated rat hearts; other FA doses were not reported by Moens *et al*. [6]. Moat *et al.* [20] also observed significant eNOS coupling starting at a dose of 5 μM in cultured endothelial cells, which was associated with improved endothelial function. Interestingly, increasing the FA concentration to 100 μM was associated with a trend towards poorer recovery with the longer duration of ischaemia (35 min) in the present study. In our *in vivo* experiments, we administered 3.3 mg FA per 100 g BW starting 10 min after the start of LAD occlusion; Moens *et al*. applied 10 mg FA per animal, also starting 10 min after the start of LAD occlusion. Assuming an animal weight of ~300 g in Moens study (rat body weights were not reported in that study), the total amount and timing of FA administered is approximately similar between both studies. Assuming that FA rapidly diffuses into the water content of the animal, with water content assumed to be 70% of body weight, the FA bolus can results in a 100 μM concentration. Our data on the isolated heart suggest that the therapeutic window for FA induced cardioprotection probably concerns a bell shaped concentration-response relation for I/R injury. A bell shaped dose-dependent cardiac response to tetrahydrybiopterin (BH4, one of the supposed cellular target of FA) was also recently observed in cardiac remodelling [21], with an optimal dose of 35 mg/kg/d. Although it is currently unclear whether FA protective effects are dependent on restoration of BH4 levels [19,22], the data do suggest that FA dosing should be carefully titrated to its optimal effect, hampering an easy translation to clinical practice.

### Folic acid protection and duration of ischaemia

We report that FA induced cardioprotection is critically dependent on ischaemic duration, with loss of FA cardioprotection with longer periods of ischaemia. Such behaviour indicates that FA protection mainly delays I/R injury without actually halting it. Other cardioprotective interventions have also shown a loss of cardioprotective potential with extension of ischaemia length [1,8,9]. It has been shown that the ischaemia-induced reduction in NOS activity is also critically dependent on the duration of ischaemia, with a sharp fall in activity after 30 min of ischaemia [23]. The drop in NOS activity could only be rescued by acute BH4 administration for ischaemic periods shorter than 30 min, probably due to irreversible oxidation of endogenous BH4. It is possible that the loss of FA protection with an ischaemic length of 35 min, as observed in the present study, is due to this irreversible loss of BH4. Further studies will be necessary to examine the role of BH4 more closely.

### Folic acid protection is mediated through eNOS

Three NOS isoforms can exist in the heart: the constitutive eNOS and nNOS and the inducible iNOS. In the present study we observed that eNOS dimerization was associated with FA cardioprotective effects, in support of the study by Moens [6]. In addition we demonstrated that eNOS was mandatory for FA cardioprotection. Thus, eNOS appears to be central to the FA cardioprotective mechanism, with dispensable roles for nNOS and iNOS. That nNOS probably is not involved is commensurate with the observation that this isoform has no effect on cardiac I/R injury [24]. iNOS is only present following an significant immunological or inflammatory stimuli that was not present in our animal models. However, it is possible that in conditions where these stimuli are present, FA may also protect through maintaining iNOS in its dimeric configuration.

### Folic acid protection in diseased animals

FA did not protect the aged or pre-diabetic hearts against cardiac infarction, whether anaesthetised with pentobarbital or fentanyl/propofol. This was somewhat surprising since it has been reported that especially diabetes and aging are associated with eNOS uncoupling, endothelial dysfunction and diminished content of tetrahydrobiopterin content [25-27]. FA is reported to enhance the binding of BH4 to eNOS [28] and increase BH4 by facilitating enzymatic reduction of its oxidized forms [29]. Although it is known that several cardioprotective interventions lose its effectiveness with aging and prolonged diabetes [9,30] due to impaired cellular signalling, FA treatment was anticipated to be effective because it targeted one of the crucial age– and diabetes-induced impairments (i.e. eNOS dysfunction). Our data suggest that targeting just one impaired step in the cardioprotective signalling cascade is insufficient, possibly due to the additional age-diabetes-induced impairment in the cardioprotective signalling cascades downstream of eNOS. Interestingly, although FA administration did not reduce cardiac infarction in the aged/pre-diabetic animals, it did improve haemodynamic parameters in these animals when anaesthetised with pentobarbital. These data suggest that haemodynamic function during an cardiac I/R insult might have a more direct relation with the molecular target (presumably eNOS) of FA as cardiac infarction. This FA-induced preservation of haemodynamic parameters following cardiac I/R was also observed in the pentobarbital-anaesthetised, healthy animals reported by [6]. The preserved haemodynamics are likely the result of preserved cardiac function following I/R due to a FA/BH4-mediated increase in eNOS functioning and therefore increased coronary flow [23]. That these improvements are primarily present in the diabetic animal is congruent with the observations that diabetes is associated with endothelial dysfunction (e.g. [31]). Further research is warranted here to elucidate the underlying mechanism of FA-induced haemodynamic preservation in these diseased models. That no protection against infarction was observed in the pentobarbital-anaesthetised aged and pre-diabetic animals may also be due to the already small infarct size under control condition for these animals, as compared to the pentobarbital-anaesthetised healthy controls (28 ± 4% and 35 ± 7% vs 74 ± 5%, for aged, pre-diabetic and young animals, respectively). It has indeed been reported that the initial, pre-diabetic state of diabetes is associated with paradoxical cardioprotection [30,32]. Interestingly, obesity also reduced infarct size in an model of insulin-insensitive rats, probably through increased AKT-eNOS phosphorylation [33]. Also for aged hearts reduced infarct size can be observed [34,35], although similar [11] or increased infarct size [9] have also been reported.

### Folic acid protection and anaesthesia

It is important that protective interventions designed preclinically to limit I/R injury are also efficacious when applied during clinically relevant anaesthetic regimens. It should be realised that cardioprotective interventions in the clinic are mostly applied during controlled perioperative conditions such as cardiac bypass surgery. Such conditions mandate the use of anesthesia, which nowadays frequently exists of fentanyl-propofol, and never pentobarbital. The present study demonstrates that acute high-dose FA treatment loses its protective potential against I/R injury in the presence of clinically relevant fentanyl/propofol anaesthesia. Moreover, FA protective effects on the haemodynamics following cardiac I/R are also lost with fentanyl/propofol anaesthesia. Total intravenous anaesthesia (TIVA), employing propofol in combination with an opioid (fentanyl), is currently one of the most popular and reliable anaesthetic regimen of choice in the clinical arena. As such, our study warrants against the development of FA therapy under clinical conditions where the patient is anaesthetised with fentanyl/propofol. Our study is in support of recent findings that remote ischaemic preconditioning also may lose its cardioprotective efficacy on a background anaesthesia of propofol [36,37]. Propofol itself has been shown to protect against I/R injury in the isolated heart, likely through an antioxidant mechanism [38,39]. In contrast, volatile anaesthetics such as isoflurane and sevoflurane generate ROS [40]. Since FA cardioprotective mechanism relates to reductions in ROS due to maintenance of eNOS dimerization, it is likely that the scavenging of ROS by propofol negates FA protective effects. Alternatively, the loss of FA cardioprotection in the young animals with fentanyl/propofol anesthesia can also result from that the heart is already in a opioid (fentanyl)-induced cardioprotective state. Indeed, our data indicate that infarct size is reduced in the young animals anaesthetized with fentanyl/propofol as compared to pentobarbital (Figure 6D versus Figure 5D). Further studies are necessary to adequately answer these questions. Pentobarbital and the volatile anaesthetics isoflurane and sevoflurane are the preferred anaesthetic substances in pre-clinical research. Also in the first report of FA protection against cardiac I/R injury animals were anaesthetised with pentobarbital [6]. Unfortunately, fentanyl/propofol is almost never used in animal research, probably because the ideal route for its delivery is intravenously, requiring additional surgical skills to execute in small animals. However, the recognition of the importance of translational science may promote the use of fentanyl/propofol in animal research. We have recently demonstrated that fentanyl/propofol can also be applied in murine research through intraperitoneal delivery, opening up the avenue of using this clinically relevant anaesthetic regimen in small animal studies [41].

### Methodological considerations

In the current study we observed reduced LDH release with an extended period of ischemia (25 min ischemia versus 35 min ischemia; Figure 1A versus Figure 2A). Other studies have sometimes also demonstrated increased injury with decreased ischemic duration [42]. For example, decreased mechanical recovery following 12 ischemia as compared to 32 min of ischaemia was reported [42]. The authors stated that this decreased recovery with shorter ischemia may be due to that at 12 min of ischemia, reperfusion is started close to the time that contracture reached its peak value, as compared to reperfusion after 32 min ischemia. Reperfusion starting after 32 min ischemia coincided with the time that the peak value of contracture is already past and contracture is rapidly diminishing. In our experiments the peak of contracture developed at 17 min of ischemia in both the 25 min I and 35 min of ischemia. Thus, reperfusion at 35 min of ischemia is already 18 min after peak contracture, whereas reperfusion at 25 min ischemia is only 8 min after peak contracture. This phenomenon may also explain the increased I/R damage with shorter ischemia observed in the current manuscript.

The cardioprotective effects of FA were examined by applying FA before, during and after ischemia in the isolated heart model, or applying FA only during the regional ischaemia in the *in vivo* condition. These protocols were specifically chosen to 1) characterise the highest cardioprotective potential with FA administration in the isolated heart, by supplying FA before, during and after ischemia in the laboratory condition, and 2) characterise FA cardioprotective potential using a clinically-relevant protocol of supplying FA only when needed (thus mimicking the situation that a patient is suddenly presented with an acute MI, i.e. ischemia is already present). In addition, using this protocol allowed direct comparison with the study of Moens et al. [6], who also provide FA only during the regional ischemic period. However, it remains to be determined whether FA administered *before* ischaemia can protect with fentanyl/propofol anaesthesia and/or the diabetic and aged hearts.

## Conclusions

In conclusion, we have demonstrated that the initial very promising cardioprotective intervention of FA treatment against cardiac I/R injury, is critically dependent on several important experimental conditions with relevance to the clinical scenario, such as concentration, ischaemic duration, anaesthetic regimen employed and health status of the animals. Extensive pre-clinical testing of potential cardioprotective interventions, with unbiased emphasis on both positive and negative results, is imperative before embarking on clinical testing.

## Abbreviations

eNOS: Endothelial nitric oxide synthase
FA: Folic acid
I/R: Ischemia-reperfusion
I: Ischemia
LDH: Lactate dehydrogenase enzyme
TTC: 2, 3, 5-triphenyltetrazolium chloride
WT: Wild-type
